# Discovering Numerical Differences between Animal and Plant microRNAs

**DOI:** 10.1371/journal.pone.0165152

**Published:** 2016-10-21

**Authors:** Rongsheng Zhu, Zhanguo Zhang, Yang Li, Zhenbang Hu, Dawei Xin, Zhaoming Qi, Qingshan Chen

**Affiliations:** 1 College of Science, Northeast Agricultural University, Harbin, China; 2 College of Agronomy, Northeast Agricultural University, Harbin, China; Harbin Medical University, CHINA

## Abstract

Previous studies have confirmed that there are many differences between animal and plant microRNAs (miRNAs), and that numerical features based on sequence and structure can be used to predict the function of individual miRNAs. However, there is little research regarding numerical differences between animal and plant miRNAs, and whether a single numerical feature or combination of features could be used to distinguish animal and plant miRNAs or not. Therefore, in current study we aimed to discover numerical features that could be used to accomplish this. We performed a large-scale analysis of 132 miRNA numerical features, and identified 17 highly significant distinguishing features. However, none of the features independently could clearly differentiate animal and plant miRNAs. By further analysis, we found a four-feature subset that included helix number, stack number, length of pre-miRNA, and minimum free energy, and developed a logistic classifier that could distinguish animal and plant miRNAs effectively. The precision of the classifier was greater than 80%. Using this tool, we confirmed that there were universal differences between animal and plant miRNAs, and that a single feature was unable to adequately distinguish the difference. This feature set and classifier represent a valuable tool for identifying differences between animal and plant miRNAs at a molecular level.

## Introduction

Plant and animal microRNAs (miRNAs) play crucial roles in developmental timing[1–10], cellular differentiation[11, 12], proliferation[13–20], apoptosis[21–26], cell identity and fate[1], and response to environmental stress[11, 12, 27], and appear to predominantly exert their influence by controlling their target genes. There are many obvious similarities between plant and animal miRNAs [28–31]. For example, their mature lengths always range from 19 to 24 nucleotides, they regulate gene expression through interactions with target mRNAs, and these targets are often involved in regulating key developmental events. However, there are also many differences [28–31]. The first step of animal miRNA biogenesis involves DROSHA nuclease, but this role is carried out by DCL1 in plants. Some animal miRNAs are generated from polycistronic transcripts located in intergenic regions of the chromosome, while others are produced from introns, whereas the majority of plant miRNAs are derived from single primary transcripts from loci found in the intergenic regions. In addition, animal miRNAs mainly act by translational repression using targets at the 3′-UTR, whereas plant miRNAs mainly regulate their targets by cleavage in the coding region of the RNA.

Recently, several studies have shown that miRNA genes are lineage-specific or species-specific, and that numerical features of miRNA genes also be conserved[32, 33]. Numerical features of miRNA genes refer to quantity index which are used to describe nucleotide content, secondary structure information, free energy and information entropy and so on. These findings imply that there may be numerical differences between animal and plant miRNAs. We therefore aimed to identify any significantly different numerical differences and explore the possibility that these differences could be used to distinguish between animal and plant miRNAs.

We selected 10951 animal and 3188 plant miRNA genes from miRBase (version21)[34] for use as a basic library and examined 132 numerical features that included sequence, structure, energy, and information entropy using the Perl program. We systematically analyzed numerical differences between animal and plant miRNAs using several statistical analysis methods. We found several numerical features, which include helix number, stack number, length of pre-miRNA, MFE and so on that could be used to differentiate between plant and animal miRNA genes. However, none of the numerical differences were sufficient on their own to clearly distinguish between individual animal and plant miRNAs. Using these results, we developed an efficient classifier to distinguish between plant and animal miRNAs based on the differences in the miRNA numerical features. Our findings demonstrate that combinations of numerical features can be used to effectively identify plant and animal miRNAs.

## Materials and Methods

### miRNAs gene sequences

We selected 10951 animal and 3188 plant miRNA genes from miRBase for use in this analysis. Details on these genes are shown in Table 1.

**Table 1:** Basic information of candidate species.

Species Class	Species Name	Number of miRNA precursor
Animal	*Ciona intestinalis*	348
Animal	*Xenopus tropicalis*	189
Animal	*Gallus gallus*	734
Animal	*Canis familiaris*	324
Animal	*Equus caballus*	341
Animal	*Monodelphis domestica*	460
Animal	*Macaca mulatta*	615
Animal	*Homo sapiens*	1872
Animal	*Pan troglodytes*	656
Animal	*Pongo pygmaeus*	634
Animal	*Ornithorhynchus anatinus*	396
Animal	*Mus musculus*	1186
Animal	*Rattus norvegicus*	449
Animal	*Bos taurus*	798
Animal	*Sus scrofa*	280
Animal	*Danio rerio*	346
Animal	*Fugu rubripes*	129
Animal	*Bombyx mori*	489
Animal	*Drosophila pseudoobscura*	210
Animal	*Caenorhabditis elegans*	223
Animal	*Capitella teleta*	124
Animal	*Schmidtea mediterranea*	148
plant	*Physcomitrella patens*	229
Plant	*Arabidopsis thaliana*	298
Plant	*Glycine max*	505
Plant	*Medicago truncatula*	672
Plant	*Populus trichocarpa*	352
Plant	*Vitis vinifera*	163
Plant	*Oryza sativa*	592
Plant	*Sorghum bicolor*	205
Plant	*Zea mays*	172

**Note:** All sequences come from miRBase database.

### Obtaining numerical features of miRNA

We extracted 132 numerical features that included sequence, structure, energy, and information entropy by designing a Perl program (S1 File). These features were divided into eight classes, and the serial numbers and names of the features are described in S1 Table. The first class referred to the frequency characteristics of single nucleotides. The second class referred to two-base combinations of the four bases A, C, G, and U, while the third class referred to three-base combinations of the four bases.

**Fig 1:**
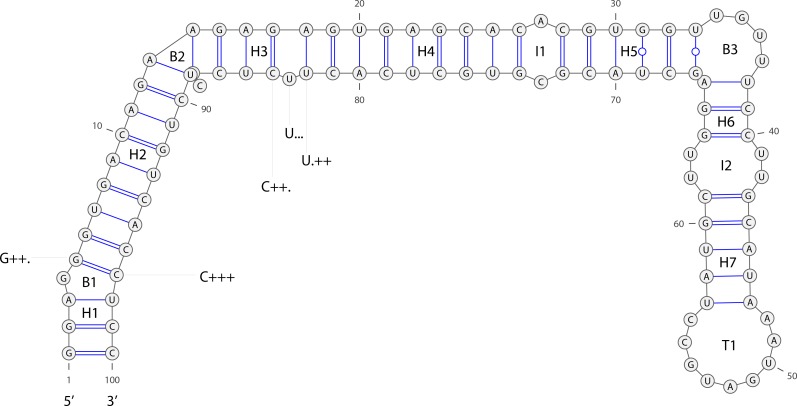
Partial numerical features of miRNA. Osa-mir156a secondary structure as predicted by Mfold. H1~H7 denote helices. I1~I2 denote interior loops. T1 denote terminal loops or hairpin loops. B1~B3 denote bulge loops. ‘G++.’ indicates that the left base of G is a matching base (‘+’ denote matching, the left base of G base corresponding to the first mark behind G) and the right base of G is mismatching base (‘.’ denote mismatching, the right base of G base corresponding to the third mark behind G). G base is a matching base (the mark of G base is the second mark behind G).

The fourth class referred to frequency features of the secondary structure matching state. Based on RNA secondary structure predicted by Mfold[35], the matching state of each nucleotide was described using the method presented by Xue et al [36]. For example, “C++.” indicates that this nucleotide at the site is "A", with a left matching site, a right mismatching site in the secondary structure and itself is a matching site. Examples are shown in Fig 1. There were 32 frequency features for the secondary structure matching state of miRNAs.

The fifth class included the length of miRNA genes, the number of bulge loops, the number of helices, the number of interior loops, and the number of stacks. Except for the length of the gene, the features were taken from Mfold predictions of secondary structure. Detailed examples are shown in Fig 1. The sixth class included the minimum free energy (MFE)[37], the adjusted MFE[38], and the MFE index[39], while the seventh class included G+C content, (G+C)/(A+U) ratio, A/C ratio, and G/U ratio.

The eighth class referred to features related to information entropy. The information entropy[40] was calculated using the formula:

$$E=-\sum_{}^{}p\log_{2}p$$ (1)

Formula (1) generated four kinds of information entropy (IE) related to the frequency of single nucleotides (IESN), dual nucleotides (IEDN), triple nucleotides (IETN), and the matching state frequency of the secondary structure (IESS). The eight classes were designated A–H in corresponding order. The p-value is frequency of every class nucleotides (For example, frequency of A, C, G and U or frequency of AA, AC, AG, AU, CA, CC, CG, CU, GA, GC, GG, GU, UA, UC, UG and UU). Formula (1) generated four kinds of entropy information related to the frequency of single nucleotides, dual nucleotides, triple nucleotides, and the matching state frequency of the secondary structure.

The 132 numerical features of 10951 animal and 3188 plant miRNA have been obtained and kept in S2 Table.

### Basic statistical analysis methods

We applied a two-sample Kolmogorov-Smirnov test[41, 42] and t-test to determine whether there were numerical differences between animal and plant miRNAs. The two-sample Kolmogorov-Smirnov test is a nonparametric test that can be used to compare two samples. The Kolmogorov-Smirnov statistic quantifies a distance between the empirical distribution functions of two samples, and is sensitive to differences in both location and shape of the empirical cumulative distribution functions of the two samples.

The Kolmogorov-Smirnov statistic for two given cumulative distribution functions F1(x) and F2(x) is shown below:

$$D=\sup|F_{1}(x)-F_{2}(x)|$$ (2)

The sup is abbreviation of the supremum of one numerical set.

### Feature selection and classification method

We applied several feature selection methods to analyze numerical features of the miRNAs, and used the selected features to build a classifier for differentiating between animal and plant miRNAs. Seven feature selection search methods were selected: BestFirst, ExhaustiveSearch, GeneticSearch, GreedyStepwise, LinearForwardSelection, RandomSearch, and RankSearch. These methods have been described previously[43]. The cfsSubsetEval and FilteredSubsetEval attribute evaluators[44] were selected, and the Logistic[45]and J48[46]models were selected as classification algorithms. NaiveBayes, BayesNet, FilteredClassifier, ZeroR, and RandomForest were used as described previously[47]. Those algorithms have been implemented by Weka [48]. About attribute evaluators, search methods and classification algorithms, we have introduced their details in S3 Table.

## Results

### Evaluating numerical differences between animal and plant miRNAs based on a single numerical feature

We used a Kolmogorov-Smirnov test and a t-test to analyze 132 numerical features in animal and plant miRNAs. Because the Kolmogorov-Smirnov test was more sensitive than the t-test, the majority of the statistical inferences were generated by the Kolmogorov-Smirnov test. The t-test was used to judge higher or lower values for every numerical feature between animal and plant miRNAs.

#### Evaluating results for 132 numerical features

**Fig 2:**
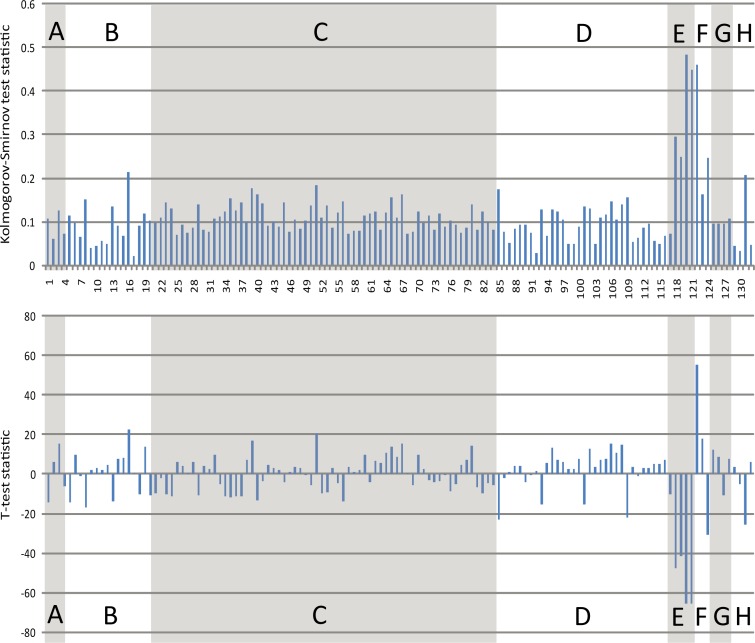
Statistical test results of differences between animal and plant miRNAs based on 132 numerical features and two test methods. The upper panel shows results of the Kolmogorov-Smirnov test, while the bottom panel shows results of t-tests. The x-axis shows the serial number of the 132 numerical features. Description of the numerical features and A–H classes are shown in S1 Table.

Results of our analyses are shown in Fig 2 and S1 Table. When the *p*-value threshold was set as 0.001, we found that there were 129 significant different features by Kolmogorov-Smirnov test, and 105 significantly different features by t-test. This demonstrated that there were universal differences between animal and plant miRNAs.

To further clarify our results, the threshold for the Kolmogorov-Smirnov test statistic was set at 0.15. Using this threshold, we selected 17 significantly different numerical features: AU%, GU%, AUC%, GAC%, GAU%, GUC%, CUC%, A…%, U…%, helix number, interior loop number, stack number, length of pre-miRNA, MFE, adjusted MFE, MFE index, and information entropy of secondary structure. Except for GU%, GAC%, GUC%, and CUC%, the results for all features were higher in plant miRNAs than in animal miRNAs.

#### Specific differences between animal and plant miRNAs based on the top three significant numerical features

**Fig 3:**
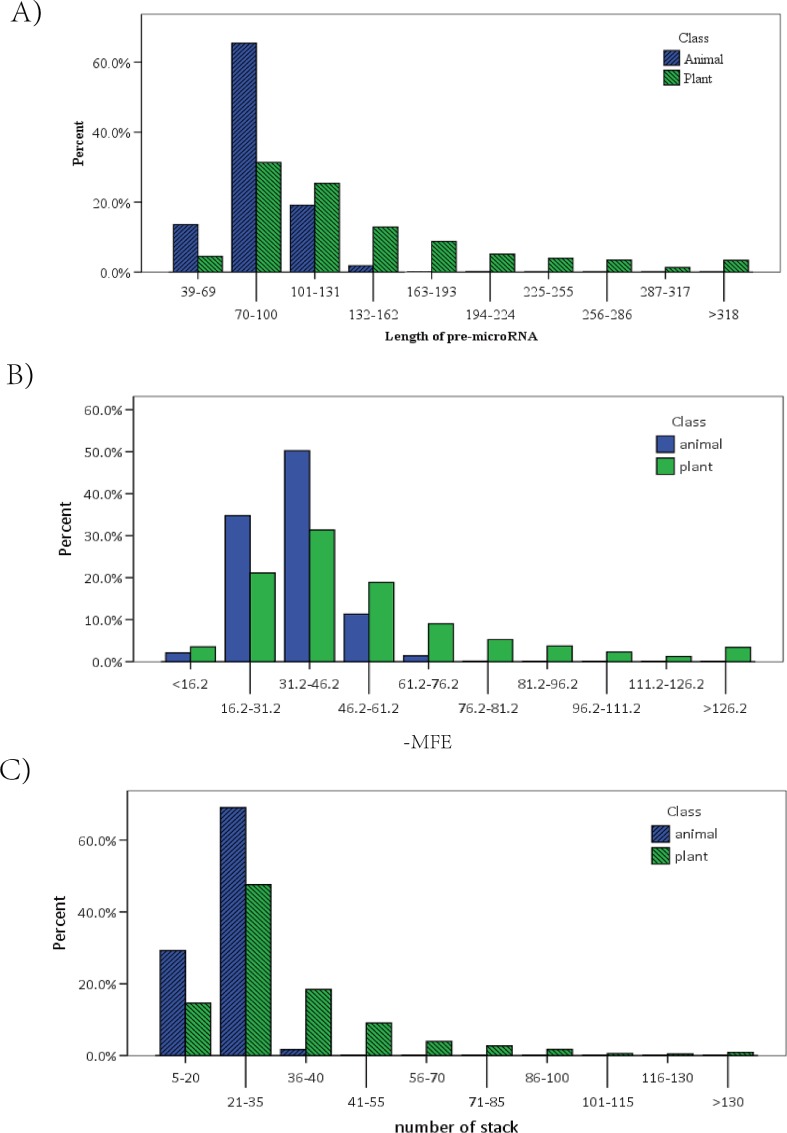
Distribution bar plot of lengths of pre-miRNAs, MFE, and number of stacks for animal and plant miRNAs. The Fig 3A is a grouping distribution map about length of pre-miRNA about animal and plant. The Fig 3B refer to MFE and the Fig 3C refer to stack number of miRNAs.

The Kolmogorov-Smirnov test statistic was much higher for three out of the 17 significantly different numerical features, specifically stack number, length of pre-miRNA, and MFE. We designed a bar plot for analyzing differences in the three features between animal and plant miRNAs in detail. Our results are shown in Fig 3.

As shown in Fig 3A, we found that the distribution of pre-miRNA length in animals was more concentrated than that observed in plants, with >65% of sequences being 70–100 nt in length. The length of plant pre-miRNA was more diverse: only 35% of plant pre-miRNAs were in the 70–100 nt range, and nearly 5% of sequences were longer than 318 nt. In contrast, there were very few animal pre-miRNAs that were longer than 160 nt.

We found that the distribution of animal miRNA MFE values was also more concentrated than that of plants, with over 85% of animal MFE values greater than −46.2 kcal (Fig 3B). Again, the MFE values for plant miRNAs were more widely distributed. Only 50% of plant miRNAs had a MFE value greater than −46.2 kcal, but nearly 4% were larger than −126.2 kcal. Few animal MFE values were less than −76.2 kcal (Fig 3B).

Fig 3C shows that distribution of animal miRNA stack numbers was highly concentrated, and over 90% of animal stack numbers were less than 35. Few animal stack numbers were higher than 40. Only 60% of plant miRNA stack numbers were less than 35, but over 20% were more than 40.

Although there were very obvious differences between animal and plant miRNAs based on these three numerical features, there was a large amount of overlap. This showed that a single feature was not sufficient for distinguishing between animal and plant miRNAs.

#### Single feature differences law for animal and plant miRNAs based on the Kolmogorov-Smirnov test statistic

**Fig 4:**
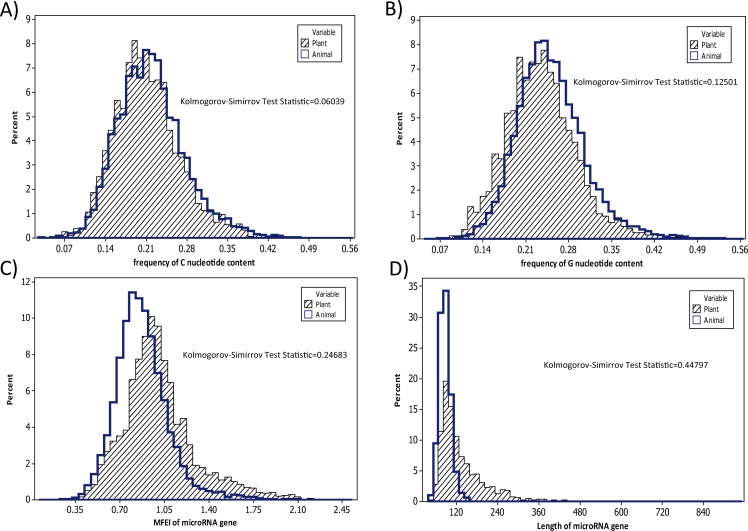
Frequency distribution plot of four numerical features of miRNAs. The C content, G content, MFE index, and length of miRNA were selected based on results of the Kolmogorov-Smirnov test statistic.

To outline a law for identifying differences between plant and animal miRNAs using a single numerical feature, we selected C%, G%, MFE index, and length of pre-miRNA. The Kolmogorov-Smirnov test statistic was from small to large. Based on these parameters, we designed a frequency density plot that included four subplots. The selected features were on four different levels based on the Kolmogorov-Smirnov test statistic. As shown in Fig 4, although feature distribution differences became clearer the closer the Kolmogorov-Smirnov test statistic became to 0.5, there was still a large area of overlap between animal and plant feature distribution density curves. This again showed that a single numerical feature was not sufficient to differentiate between animal and plant miRNAs. In general, we found that the larger the value of the Kolmogorov-Smirnov test statistic, the more significant the difference between the animal and plant miRNA numerical feature. As a result of these findings, we decided to evaluate a combination of features to try to distinguish between plant and animal miRNAs.

### Identification of feature sets that could be used to differentiate between animal and plant miRNAs

Based on the Kolmogorov-Smirnov test statistic values, the top 17 out of the 132 examined numerical features were selected. We applied a feature selection technique for these 17 significantly different features, including two attribute evaluators, CfsSubsetEval and FilteredSubsetEval, and six search methods, BestFirst, ExhaustiveSearch, GeneticSearch, GreedyStepwise, LinearForwardSelection, and RandomSearch. The analysis was finished by Weka software[48]. Our analysis results are shown in Table 2.

**Table 2:** Results of features selection.

Attribute Evaluator	Search Method	Selected Feature
CfsSubsetEval	BestFirst	118,120,121,122
CfsSubsetEval	ExhaustiveSearch	118,120,121,122
CfsSubsetEval	GeneticSearch	51,118,120,121,122
FilteredSubsetEval	GreedyStepwise	118,120,121,122
FilteredSubsetEval	LinearForwardSelection	118,120,121,122
FilteredSubsetEval	RandomSearch	120,121,122

Note: Serial number information of being selected features refer to S1 Table. The number 51 represent GUC content frequency, the number 118 represent number of helix, the number 120 represent number of stack, the number 121 represent length of hairpin and the number 122 represent minimum free energy of pre-miRNA’s secondary structure. Attribute Evaluator and Search Method refer to papers[43, 44] and all details have been recorded in S3 Table.

From the results, we found that four out of the 17 numerical features almost always arose in the six feature selection strategies. They were helix number, stack number, length of pre-miRNA, and MFE. Therefore, this feature subset was used as the basis of the classifier.

### Building a classifier for animal and plant miRNAs

We applied seven classifiers for two feature subsets. Our analysis results are shown in Table 3. The seven classifiers included NaiveBayes, BayesNet, Logistic, FilteredClassifier, ZeroR, J48, and RandomForest. The S1 feature subset included the four features identified by feature selection, while the S2 feature subset included all 17 significantly different features. Analysis was performed using Weka software.

**Table 3:** Results of evaluation based on different classifiers.

Classifier	Sample Set	TP Rate	Precision	Recall	ROC Area
NaiveBayes	S1	0.849	0.843	0.849	0.773
BayesNet	S1	0.843	0.833	0.843	0.801
Logistic	S1	0.854	0.854	0.835	0.805
FilteredClassifier	S1	0.856	0.856	0.856	0.789
ZeroR	S1	0.772	0.595	0.772	0.5
J48	S1	0.855	0.851	0.855	0.764
RandomForest	S1	0.815	0.804	0.815	0.744
NaiveBayes	S2	0.844	0.835	0.844	0.795
BayesNet	S2	0.84	0.833	0.84	0.807
Logistic	S2	0.86	0.861	0.86	0.816
FilteredClassifier	S2	0.861	0.86	0.861	0.778
ZeroR	S2	0.772	0.595	0.772	0.5
J48	S2	0.862	0.857	0.862	0.759
RandomForest	S2	0.836	0.827	0.836	0.77

Note: 10-fold cross-validation; S1 include the helix, stack number, length and MFE; S2 include AU, GU, AUC, GAC, GAU, GUC, CUC, A…, U…, helix number, interior loop number, stack number, length of pre-miRNA, MFE, AMFE, MFEI and IESS.

As shown in Table 3, we found that the maximum receiver operating characteristic (ROC) areas for each classifier all occurred in the logistic model for both of the feature subsets. For S1, the logistic classifier's ROC area was 0.805, and the precision of classification was 0.854. For S2, the logistic classifier's ROC area was 0.816, with a precision of classification of 0.861. The performance of the classifiers was very similar based on the two feature subsets. Consistent with our aim of determining the smallest number of numerical features that could be used to differentiate between animal and plant miRNAs, S1 and the logistic classifier were selected as our research model. The logistic model was as follows:

$$Logit(P)=6.1436+0.0893x_{1}-0.0691x_{2}-0.0241x_{3}+0.0263x_{4}$$ (3)

Where P stands for probability of animal miRNA, x_1_ denotes helix number, x_2_ denotes stack number, x_3_ denotes length of pre-miRNA; and x_4_ denotes MFE. The model and its coefficients were all significant (*P* = 0.01).

## Discussion

Although there were significant differences between animal and plant miRNAs based on each of the 17 numerical features, none of them could be used in isolation to reliably assess miRNAs. Therefore, a feature selection and classifier method was applied, and a feature subset and analysis model were obtained. We could distinguish between animal and plant miRNAs using the logistic model that was built based on four numerical features. Candidate miRNAs analyzed for these four features, specifically helix number, stack number, length of pre-miRNA, and MFE, could be classified with >85% precision.

Interestingly, 13 of 17 significantly different numerical features were higher in plant miRNAs than in animal miRNAs. We speculated that there may be were more complexity and a larger variety of sequences and structures in plant miRNAs compared with those in animals[29].

The selected feature subset was composed of the top four features based on Kolmogorov-Smirnov test statistic values. The larger the Kolmogorov-Smirnov test statistic value the more significant the difference between animal and plant miRNAs for a certain numerical feature. This relationship is shown in Fig 4. To clarify this relationship between Kolmogorov-Smirnov test statistic value and the detailed numerical difference between animal and plant miRNA, we used stack number of miRNAs as an example. The results of this analysis are shown in S1 Fig. Based on the results shown in Fig 4 and S1 Fig, we determined that the Kolmogorov-Smirnov test statistic value could be used as an evaluation criterion for differences in frequency distribution.

In this study, several feature selection methods were applied and a high level of accuracy was obtained. However, the relationship among features was not considered. To determine whether a relationship existed between the features, we calculated the Pearson correlation coefficients between any two features (S2 Fig). This analysis showed that relationships between features were ubiquitous, and therefore the nature of a feature relationship might influence the results of feature selection. Feature transformation may be a good method for obtaining effective features for classification without such bias.

By our analysis, 17 highly significant distinguishing features were identified and they would become main numerical difference between plant and animal miRNAs. By further analysis, we found a four-feature subset that included helix number, stack number, length of pre-miRNA, and minimum free energy, and developed a logistic classifier that could distinguish animal and plant miRNAs effectively. The precision of the classifier was greater than 80%. Using this tool, we confirmed that there were universal differences between animal and plant miRNAs, and that a single feature was unable to adequately distinguish the difference. This feature set and classifier represent a valuable tool for identifying between animal and plant miRNAs at a molecular level.

## Supporting Information

S1 Fig Sketch map of distributions of animal and plant miRNAs based on stack number. (A) Marked empirical distribution function of stack number for animal and plant miRNAs. (B) Marked frequency distribution of stack number for animal and plant siRNAs. (C) Marked frequency distribution of stack number based on boxed area shown in (B).
(PDF)

S2 Fig Color map of correlation coefficients between any two numerical features of the miRNAs. (PDF)

S1 File A Perl script for obtaining numerical features of miRNAs. (PL)

S1 Table Statistical test results of differences between animal and plant miRNAs based on 132 numerical features and two test methods. (XLSX)

S2 Table The 132 numerical features of 10951 animal and 3188 plant miRNA. (XLSX)

S3 Table Detail description of attribute evaluators, search methods and classification algorithms Program. (XLSX)

## References

[1] Abbott AL, Alvarez-Saavedra E, Miska EA, Lau NC, Bartel DP, Horvitz HR, et al The let-7 MicroRNA family members mir-48, mir-84, and mir-241 function together to regulate developmental timing in Caenorhabditis elegans. Dev Cell. 2005;9(3):403–14. 10.1016/j.devcel.2005.07.009 16139228 PMC3969732

[2] Aukerman MJ, Sakai H. Regulation of flowering time and floral organ identity by a MicroRNA and its APETALA2-like target genes. Plant Cell. 2003;15(11):2730–41. 10.1105/tpc.016238 14555699 PMC280575

[3] Boehm M, Slack F. A developmental timing microRNA and its target regulate life span in C. elegans. Science. 2005;310(5756):1954–7. 10.1126/science.1115596 . 16373574

[4] Candela H, Johnston R, Gerhold A, Foster T, Hake S. The milkweed pod1 gene encodes a KANADI protein that is required for abaxial/adaxial patterning in maize leaves. The Plant Cell. 2008;20(8):2073–87. 10.1105/tpc.108.059709 18757553 PMC2553616

[5] Cho SH, Coruh C, Axtell MJ. miR156 and miR390 regulate tasiRNA accumulation and developmental timing in Physcomitrella patens. The plant cell. 2012;24(12):4837–49. 10.1105/tpc.112.103176 23263766 PMC3556961

[6] Jung J-H, Seo PJ, Ahn JH, Park C-M. Arabidopsis RNA-binding protein FCA regulates microRNA172 processing in thermosensory flowering. Journal of Biological Chemistry. 2012;287(19):16007–16. 10.1074/jbc.M111.337485 22431732 PMC3346135

[7] Jung J-H, Seo PJ, Kang SK, Park C-M. miR172 signals are incorporated into the miR156 signaling pathway at the SPL3/4/5 genes in Arabidopsis developmental transitions. Plant molecular biology. 2011;76(1–2):35–45. 10.1007/s11103-011-9759-z 21373962

[8] Li S, Yang X, Wu F, He Y. HYL1 controls the miR156-mediated juvenile phase of vegetative growth. Journal of experimental botany. 2012;63(7):2787–98. 10.1093/jxb/err465 22268150 PMC3346236

[9] Wu G, Park MY, Conway SR, Wang J-W, Weigel D, Poethig RS. The sequential action of miR156 and miR172 regulates developmental timing in Arabidopsis. Cell. 2009;138(4):750–9. 10.1016/j.cell.2009.06.031 19703400 PMC2732587

[10] Yang L, Conway SR, Poethig RS. Vegetative phase change is mediated by a leaf-derived signal that represses the transcription of miR156. Development. 2011;138(2):245–9. 10.1242/dev.058578 21148189 PMC3005601

[11] Bentwich I. A postulated role for microRNA in cellular differentiation. The FASEB journal. 2005;19(8):875–9. 10.1096/fj.04-3609hyp 15923397

[12] Onnis A, Navari M, Antonicelli G, Morettini F, Mannucci S, De Falco G, et al Epstein-Barr nuclear antigen 1 induces expression of the cellular microRNA hsa-miR-127 and impairing B-cell differentiation in EBV-infected memory B cells. New insights into the pathogenesis of Burkitt lymphoma. Blood cancer journal. 2012;2(8):e84. 22941339 10.1038/bcj.2012.29 PMC3432484

[13] Brennecke J, Hipfner DR, Stark A, Russell RB, Cohen SM. bantam encodes a developmentally regulated microRNA that controls cell proliferation and regulates the proapoptotic gene hid in Drosophila. Cell. 2003;113(1):25–36. 12679032 10.1016/s0092-8674(03)00231-9

[14] Chen J-F, Mandel EM, Thomson JM, Wu Q, Callis TE, Hammond SM, et al The role of microRNA-1 and microRNA-133 in skeletal muscle proliferation and differentiation. Nature genetics. 2006;38(2):228–33. 10.1038/ng1725 16380711 PMC2538576

[15] Johnson CD, Esquela-Kerscher A, Stefani G, Byrom M, Kelnar K, Ovcharenko D, et al The let-7 microRNA represses cell proliferation pathways in human cells. Cancer research. 2007;67(16):7713–22. 10.1158/0008-5472.CAN-07-1083 17699775

[16] Lee K-H, Goan Y-G, Hsiao M, Lee C-H, Jian S-H, Lin J-T, et al MicroRNA-373 (miR-373) post-transcriptionally regulates large tumor suppressor, homolog 2 (LATS2) and stimulates proliferation in human esophageal cancer. Experimental cell research. 2009;315(15):2529–38. 10.1016/j.yexcr.2009.06.001 19501585

[17] Chen J, Feilotter HE, Paré GC, Zhang X, Pemberton JG, Garady C, et al MicroRNA-193b represses cell proliferation and regulates cyclin D1 in melanoma. The American journal of pathology. 2010;176(5):2520–9. 10.2353/ajpath.2010.091061 20304954 PMC2861116

[18] Afanasyeva EA, Mestdagh P, Kumps C, Vandesompele J, Ehemann V, Theissen J, et al MicroRNA miR-885-5p targets CDK2 and MCM5, activates p53 and inhibits proliferation and survival. Cell Death & Differentiation. 2011;18(6):974–84. 10.1038/cdd.2010.164 PMC3131937 21233845

[19] Bukhari SIA, Vasquez-Rifo A, Gagné D, Paquet ER, Zetka M, Robert C, et al The microRNA pathway controls germ cell proliferation and differentiation in C. elegans. Cell research. 2012;22(6):1034–45. 10.1038/cr.2012.31 22370633 PMC3367518

[20] Liu XS, Chopp M, Wang XL, Zhang L, Hozeska-Solgot A, Tang T, et al MicroRNA-17-92 cluster mediates the proliferation and survival of neural progenitor cells after stroke. Journal of Biological Chemistry. 2013;288(18):12478–88. 10.1074/jbc.M112.449025 23511639 PMC3642296

[21] Thompson BJ, Cohen SM. The Hippo pathway regulates the bantam microRNA to control cell proliferation and apoptosis in Drosophila. Cell. 2006;126(4):767–74. 10.1016/j.cell.2006.07.013 16923395

[22] Chen Y, Stallings RL. Differential patterns of microRNA expression in neuroblastoma are correlated with prognosis, differentiation, and apoptosis. Cancer research. 2007;67(3):976–83. 10.1158/0008-5472.CAN-06-3667 17283129

[23] Jaklevic B, Uyetake L, Wichmann A, Bilak A, English CN, Su TT. Modulation of ionizing radiation-induced apoptosis by bantam microRNA in Drosophila. Developmental biology. 2008;320(1):122–30. 10.1016/j.ydbio.2008.04.043 18550049 PMC2629399

[24] Wang Y, Lee CG. MicroRNA and cancer–focus on apoptosis. Journal of cellular and molecular medicine. 2009;13(1):12–23. 10.1111/j.1582-4934.2008.00510.x 19175697 PMC3823033

[25] Buscaglia LEB, Li Y. Apoptosis and the target genes of miR-21. Chinese journal of cancer. 2011;30(6):371 10.5732/cjc.011.10132 21627859 PMC3319771

[26] Liu L, Zhang G, Liang Z, Liu X, Li T, Fan J, et al MicroRNA-15b enhances hypoxia/reoxygenation-induced apoptosis of cardiomyocytes via a mitochondrial apoptotic pathway. Apoptosis. 2014;19(1):19–29. 10.1007/s10495-013-0899-2 24043355

[27] Zhang H, Fire AZ. Cell autonomous specification of temporal identity by Caenorhabditis elegans microRNA lin-4. Developmental biology. 2010;344(2):603–10. 10.1016/j.ydbio.2010.05.018 20493184 PMC2914126

[28] Carrington JC, Ambros V. Role of microRNAs in plant and animal development. Science. 2003;301(5631):336–8. 10.1126/science.1085242 12869753

[29] Millar AA, Waterhouse PM. Plant and animal microRNAs: similarities and differences. Functional & integrative genomics. 2005;5(3):129–35. 15875226 10.1007/s10142-005-0145-2

[30] Wheeler G, Valoczi A, Havelda Z, Dalmay T. In situ detection of animal and plant microRNAs. DNA and cell biology. 2007;26(4):251–5. 10.1089/dna.2006.0538 17465891

[31] Tang G, Yan J, Gu Y, Qiao M, Fan R, Mao Y, et al Construction of short tandem target mimic (STTM) to block the functions of plant and animal microRNAs. Methods. 2012;58(2):118–25. 10.1016/j.ymeth.2012.10.006 23098881 PMC3631596

[32] Axtell MJ, Westholm JO, Lai EC. Vive la différence: biogenesis and evolution of microRNAs in plants and animals. Genome biology. 2011;12(4):1. 10.1186/gb-2011-12-4-221 PMC3218855 21554756

[33] Zhu R, Li X, Chen Q. Discovering numerical laws of plant microRNA by evolution. Biochemical and biophysical research communications. 2011;415(2):313–8. 10.1016/j.bbrc.2011.10.051 22033408

[34] Griffiths‐Jones S. miRBase: microRNA sequences and annotation. Current protocols in bioinformatics. 2010:12.9. 1-.9. 0. 10.1002/0471250953.bi1209s29 20205188

[35] Zuker M. Mfold web server for nucleic acid folding and hybridization prediction. Nucleic acids research. 2003;31(13):3406–15. 12824337 10.1093/nar/gkg595 PMC169194

[36] Xue C, Li F, He T, Liu G-P, Li Y, Zhang X. Classification of real and pseudo microRNA precursors using local structure-sequence features and support vector machine. BMC bioinformatics. 2005;6(1):1. 16381612 10.1186/1471-2105-6-310 PMC1360673

[37] Zhang B, Pan X, Cox S, Cobb G, Anderson T. Evidence that miRNAs are different from other RNAs. Cellular and Molecular Life Sciences CMLS. 2006;63(2):246–54. 10.1007/s00018-005-5467-7 16395542 PMC11136112

[38] Bonnet E, Wuyts J, Rouzé P, Van de Peer Y. Evidence that microRNA precursors, unlike other non-coding RNAs, have lower folding free energies than random sequences. Bioinformatics. 2004;20(17):2911–7. 10.1093/bioinformatics/bth374 15217813

[39] Freyhult E, Gardner PP, Moulton V. A comparison of RNA folding measures. BMC bioinformatics. 2005;6(1):1. 16202126 10.1186/1471-2105-6-241 PMC1274297

[40] Shannon CE. A mathematical theory of communication. ACM SIGMOBILE Mobile Computing and Communications Review. 2001;5(1):3–55.

[41] Massey FJ Jr. The Kolmogorov-Smirnov test for goodness of fit. Journal of the American statistical Association. 1951;46(253):68–78.

[42] Stephens MA. Use of the Kolmogorov-Smirnov, Cramér-Von Mises and related statistics without extensive tables. Journal of the Royal Statistical Society Series B (Methodological). 1970:115–22.

[43] Pearl J. Heuristics: intelligent search strategies for computer problem solving. 1984.

[44] Guyon I, Elisseeff A. An introduction to variable and feature selection. Journal of machine learning research. 2003;3(Mar):1157–82.

[45] Staskiewicz G, Czekajska-Chehab E, Uhlig S, Przegalinski J, Maciejewski R, Drop A. Logistic regression model for identification of right ventricular dysfunction in patients with acute pulmonary embolism by means of computed tomography. European journal of radiology. 2013;82(8):1236–9. 10.1016/j.ejrad.2013.02.004 23473781

[46] Schietgat L, Vens C, Struyf J, Blockeel H, Kocev D, Džeroski S. Predicting gene function using hierarchical multi-label decision tree ensembles. BMC bioinformatics. 2010;11(1):1. 20044933 10.1186/1471-2105-11-2 PMC2824675

[47] Piper ME, Loh W-Y, Smith SS, Japuntich SJ, Baker TB. Using decision tree analysis to identify risk factors for relapse to smoking. Substance use & misuse. 2011;46(4):492–510. 20397871 10.3109/10826081003682222 PMC2908723

[48] Hall M, Frank E, Holmes G, Pfahringer B, Reutemann P, Witten IH. The WEKA data mining software: an update. ACM SIGKDD explorations newsletter. 2009;11(1):10–8.

