# Supplementary figures and images for: Discovering Numerical Differences between Animal and Plant microRNAs
Source: PLoS One. 2016 Oct 21;11(10):e0165152. doi: 10.1371/journal.pone.0165152 (PMC5074594; doi:10.1371/journal.pone.0165152)

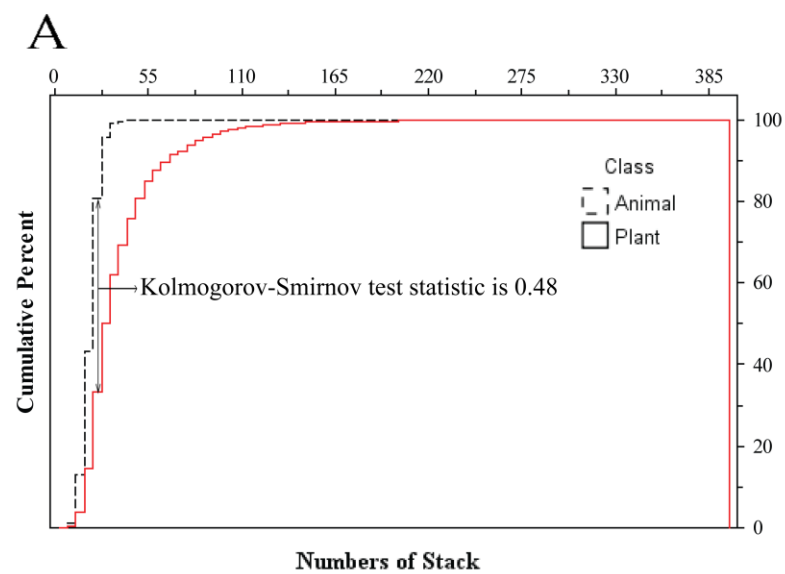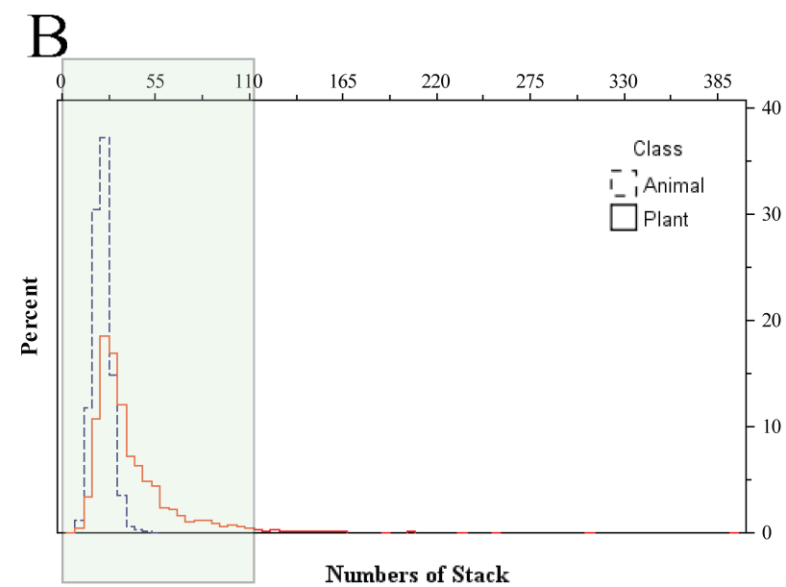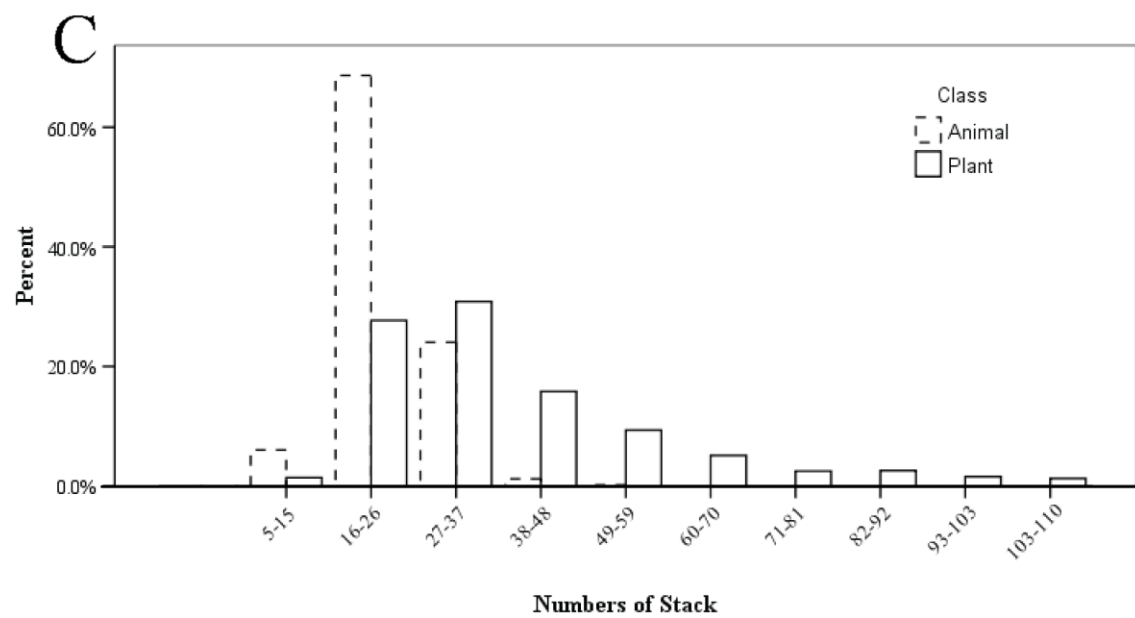

Supplement: S1 Fig — (A) Marked empirical distribution function of stack number for animal and plant miRNAs. (B) Marked frequency distribution of stack number for animal and plant siRNAs. (C) Marked frequency distribution of stack number based on boxed area shown in (B). (PDF) [file pone.0165152.s001.pdf]

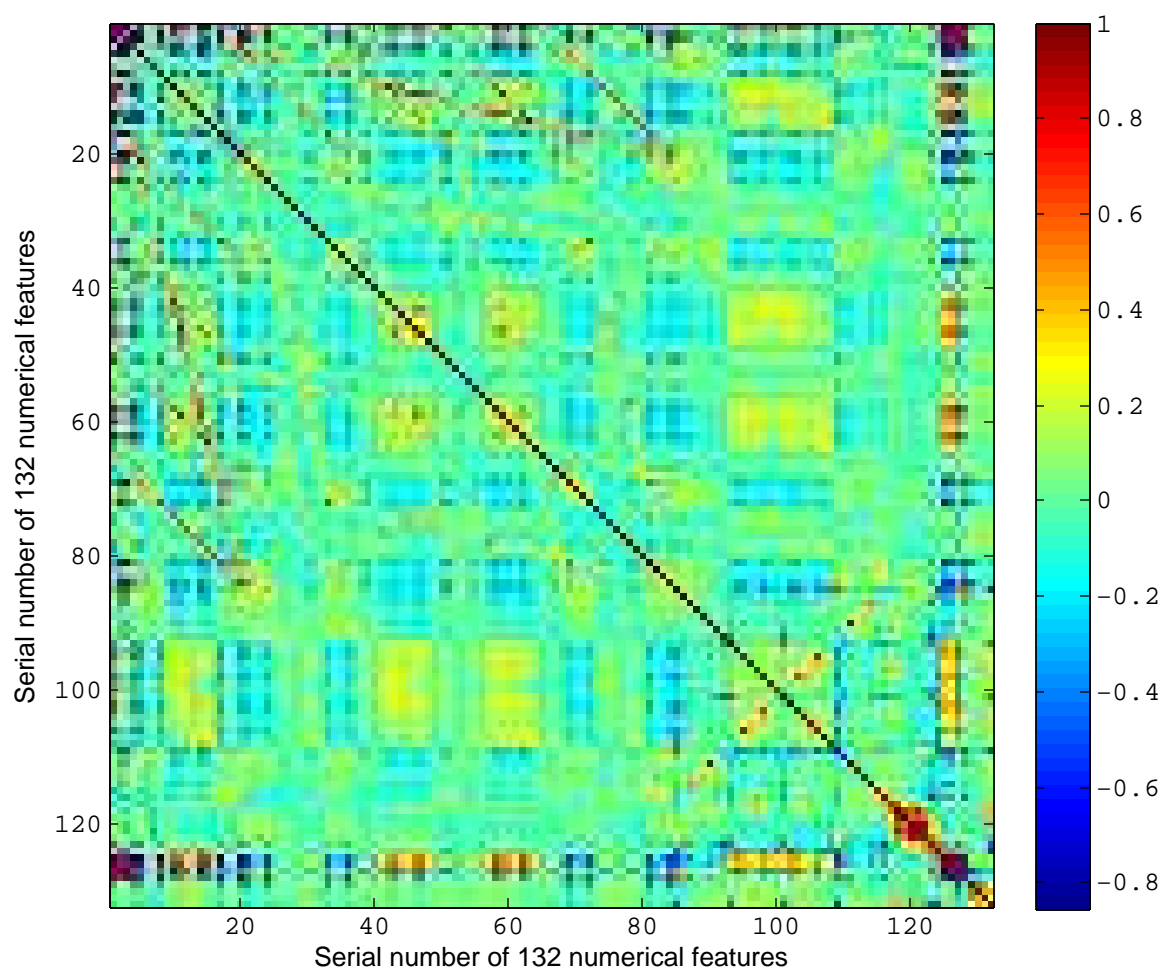

Supplement: S2 Fig — (PDF) [file pone.0165152.s002.pdf]
